# Computing and interpreting the Number Needed to Treat for Cardiovascular Outcomes Trials: Perspective on GLP-1 RA and SGLT-2i therapies

**DOI:** 10.1186/s12933-020-01034-3

**Published:** 2020-05-13

**Authors:** Lisa Ludwig, Patrice Darmon, Bruno Guerci

**Affiliations:** 1 Endocrinology, Diabetology & Nutrition, CHRU of Nancy, Brabois Hospital, Lorraine University, 54500 Vandoeuvre-lès-Nancy, France; 2 Endocrinology, Metabolic Diseases and Nutrition Department, Assistance Publique Hôpitaux de Marseille, Marseille, France; 3 INSERM, INRA, C2VN, Aix Marseille University, 13005 Marseille, France

**Keywords:** Cardiovascular Outcome Trial, Type 2 diabetes, Number Needed to Treat, GLP-1 receptor agonist, SGLT-2 inhibitors

## Abstract

The recent results of Cardiovascular Outcomes Trials (CVOTs) in type 2 diabetes have clearly established the cardiovascular (CV) safety or even the benefit of two therapeutic classes, Glucagon-Like Peptide-1 receptor agonists (GLP-1 RA) and Sodium-Glucose Co-Transporter-2 inhibitors (SGLT-2i). Publication of the latest CVOTs for these therapeutic classes also led to an update of ESC guidelines and ADA/EASD consensus report in 2019, which considers using GLP-1 RA or SGLT-2i with proven cardiovascular benefit early in the management of type 2 diabetic patient with established cardiovascular disease (CVD) or at high risk of atherosclerotic CVD. The main beneficial results of these time-to event studies are supported by conventional statistical measures attesting the effectiveness of GLP-1 RA or SGLT2i on cardiovascular events (absolute risk, absolute risk difference, relative risk, relative risk reduction, odds ratio, hazard ratio). In addition, another measure whose clinical meaning appears to be easier, the Number Needed to Treat (NNT), is often mentioned while discussing the results of CVOTs, in order to estimating the clinical utility of each drug or sometimes trying to establish a power ranking. While the value of the measure is admittedly of interest, the subtleties of its computation in time-to-event studies are little known. We provide in this article a clear and practical explanation on NNT computation methods that should be used in order to estimate its value, according to the type of study design and variables available to describe the event of interest, in any randomized controlled trial. More specifically, a focus is made on time-to-event studies of which CVOTs are part, first to describe in detail an appropriate and adjusted method of NNT computation and second to help properly interpreting NNTs with the example of CVOTs conducted with GLP-1 RA and SGLT-2i. We particularly discuss the risk of misunderstanding of NNT values in CVOTs when some specific parameters inherent in each study are not taken into account, and the following risk of erroneous comparison between NNTs across studies. The present paper highlights the importance of understanding rightfully NNTs from CVOTs and their clinical impact to get the full picture of a drug’s effectiveness.

## Background

During the last decade, Cardiovascular Outcomes Trials (CVOTs) have aroused considerable interest among diabetologists and cardiologists. In these randomized, controlled, time-to-event studies evaluating the cardiovascular (CV) safety of emerging antidiabetic drugs, the primary endpoint is most often a composite CV criterion called “3P-MACE” combining mortality from CV cause, non-fatal myocardial infarction and non-fatal stroke. CVOTs have notably clearly established the CV safety or even the benefit of two therapeutic classes, Glucagon-Like Peptide-1 receptor agonists (GLP-1 RA) and sodium-glucose co-transporter-2 inhibitors (SGLT-2i), and have positioned them as priority options in the management of type 2 diabetes, especially in patients with an established CV disease, heart failure and/or chronic kidney disease. The results of these CVOTs, confirmed by two recent meta-analyzes [1, 2] have led to the drafting of a new ADA/EASD consensus in 2018 [3], then revised at the end of 2019 [4], and to recommendations from the European Cardiology Society in 2019 [5]. The question then arises of the relative effectiveness of these molecules or classes. Indeed, the significant results obtained in the CVOTs question whether a ranking of molecules or classes could be established. In addition to the conventional statistical measures attesting the effectiveness of GLP-1 RA or SGLT2i in CVOTs (absolute risk and absolute risk difference, relative risk and relative risk reduction, odds ratio and hazard ratio), one may also refer to another measure whose clinical meaning appears to be easier: the Number Needed to Treat (NNT).

The NNT is a now common, statistical measure of the clinical utility of a treatment. After each reporting of CVOT results, a lively discussion ensued on the associated NNT and its impact, to a greater or lesser extent, on clinical practices or even on the efficiency of the drug itself. While the value of the measure is admittedly of interest, its computation remains controversial. Indeed, the seemingly simple calculation of the NNT must, however, consider some subtleties when derived from interventional studies where the occurrence of the primary endpoint is a function of time. Several methods to adjust the computation according to the design and assumptions of each study have been proposed by statisticians, some more consensual than others. Nevertheless, numerous calculation errors still occur, especially while reporting NNTs in these time-to-event studies, and of which CVOTs are an example. Indeed, a meta-analysis carried out on articles published in 4 major international journals (BMJ, JAMA, N Engl J Med, Lancet) between 2003 and 2005, and reporting results of randomized controlled trial (RCT) with a time-dependent main outcome highlighted that at least half of them reported incorrect NNT values because of an inappropriate computation method [6]. A second survey carried out in 2009 on the same journals reported 60% computation errors for studies where the occurrence of the event of interest is time-dependent [7]. The same issue, regarding complexity of NNTs’ computation and interpretation, was found within more recent publication papers discussing data from other cardioprotective drug classes such as antiplatelet therapy, Proprotein Convertase subtilsin-kexin type 9 (PCSK-9) inhibitors, or angiotensin receptor–neprilysin inhibitor (ARNI), among others [8–11]. Hence, a wide range of therapeutic areas are concerned by the issue, which emphasizes the importance of paying careful attention to the analysis of NNTs.

This article aims firstly at clarifying in a practical manner how to calculate an NNT for time-to-event RCTs while avoiding some of the classical mistakes, and secondly at helping to interpret appropriately NNTs with the example of CVOTs results. One should not expect the development or demonstration of complex statistical models, but rather a key to simple computation and interpretation of NNTs in CVOTs. Also, data from observational studies, cohorts, case–control studies or even meta-analyzes are subject to specific NNT calculation methods and will not be addressed hereafter.

## NNT computation methods

Introduced in 1988 by Laupacis et al., the NNT represents the number of patients to be treated during a given period of time to prevent the occurrence of one additional negative or unfavorable event [12]. A NNT can be calculated if the outcome of interest is binary, but unfortunately not if the main outcome associated data are continuous. The choice of the NNT computation method will depend in particular on the design of the study as well as on the type of variables describing the event of interest.

1. Computation of the absolute risk difference

**Fig. 1:**
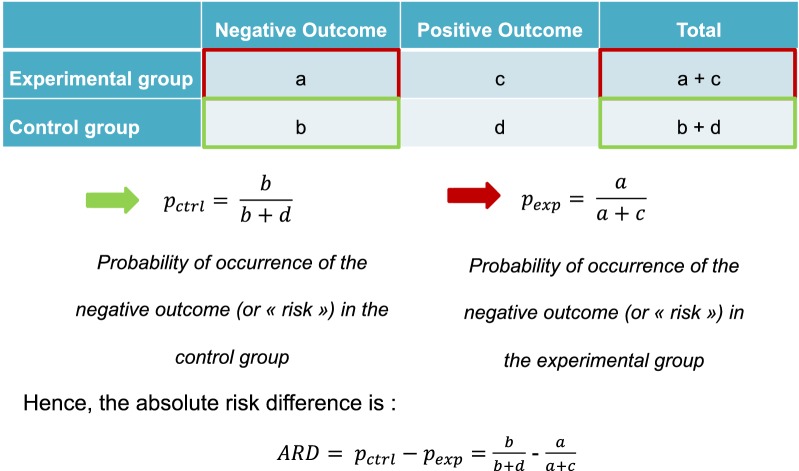
Calculation of the absolute risk difference in practice. ARD: absolute risk difference

First of all, before considering the NNT calculation, the absolute risk difference needs to be computed (Fig. 1) [13]. There are two possibilities:

First, consider the probability p of occurrence of a negative event in each group (also called “risk”), for example severe hypoglycemia. If the treatment is effective, the risk of a severe hypoglycemia should be lower in the experimental group as compared to the control group. The absolute risk difference (ARD) will be as follows: ARD = p_ctrl_ – p_exp_. This is the most common case.

Then, consider a positive outcome of interest, e.g. proportion of patients free of retinopathy after 3 years of treatment. Its probability of occurrence p will rather be considered as a chance. In this case, if the treatment is effective, a greater proportion of patients should remain free of retinopathy, and thus the occurrence probability of the event should be higher in the treatment group as compared to the control group. The absolute risk difference formula should be inverted to obtain in this case: ARD = p_exp_ – p_ctrl_.

2. Binary outcome study with fixed and constant follow-up period

In RCT where the observed variables are binary (“event” vs “no event”) and all the patients are followed for a predefined period of time, the risk that is the proportion of patients who present with the unfavorable event, is measured in each group [13]. The NNT can then be estimated according to the simple proportion formula as:

$$NNT = \frac{1}{ARD}$$

with:

- ARD: the absolute risk difference corresponding to:
  - ARD = p_ctrl_ – p_exp_ if the event of interest is unfavorable
  - ARD = p_exp_ – p_ctrl_ if the event of interest is favorable
- p_ctrl_: the risk or occurrence probability of the event of interest in the control group
- p_exp_: the risk or occurrence probability of the event of interest in the experimental group.

The more effective the treatment is, the greater the absolute risk difference will be, which in turn will translates in a lower NNT. A utopian goal would be to seek an NNT of 1: treatment would prevent the occurrence of the unfavorable event in all patients. On the opposite, if a treatment had no beneficial effect on the event of interest, the absolute risk difference would be close to zero, and therefore the NNT would be infinite: even if an infinite number of patients were treated, no beneficial effect on the event of interest would be observed. Finally, a negative value of NNT, which is a dystopia for clinical practice, should drive the clinician to consider the treatment as harmful for patients. In this case, one speaks of the Number Needed to Harm (NNH) as opposed to the Number Needed to Benefit (NNB) [14].

Take the example of the EXPLORER study (RCT), which aimed to assess the effect on wound closure of a new type of dressing (sucrose octasulfate) versus a control dressing in patients with a diabetic foot ulcer [15]. The primary endpoint was the proportion of patients with a closed wound at week 20. Forty-eight percent of patients in the experimental group had a closed wound at 20 weeks compared to 30% in the control group (ARD 18%, 95% CI 5–30). Wound closure is obviously beneficial to the patient, hence:

$$NNT = \frac{1}{0.48 - 0.30} = 6.$$

In addition, NNTs should always be presented with their confidence intervals (CI), but this is rarely done [14]. To this end, one should apply the same formula to the inverted bounds of the 95% CI of the absolute risk difference (in our example 95% CI 5; 30) in order to compute the 95% CI of the NNT:

$$95\% \;CI\left( {NNT} \right) = \left[ { \frac{1}{0.30};\frac{1}{0.05} } \right] \leftrightarrow \left[ {4;20} \right]$$

After a median follow-up of 20 weeks, 6 patients (95% CI 4–20) would have to be treated with the new type of dressing to allow for one wound closure. NB: in this article, NNT will systematically be rounded upwards.

3. Time-to-event study with varying follow-up periods

In certain randomized controlled studies, the occurrence of the main endpoint is dependent on the duration of follow-up of each patient: these are referred to as *time*-*to*-*event* studies (“*the time before the event*”). One of the most evident examples of this type of time-dependent outcome may be the assessment of the effect of one specific intervention on survival or event-free survival. CVOTs are the best examples of these time-to-event studies. Indeed, the main outcome in CVOTs is often a 3P-MACE (a composite CV criterion combining mortality from CV cause, non-fatal myocardial infarction and non-fatal stroke), whose occurrence can’t be predicted or controlled, and will eventually occur at a different time point for each patient. Hence, time-to-event studies do not allow to predefine a priori a duration of patient follow-up. Rather, methodologists will pre-specify a number of events to be achieved for the study to reach the required power and statistical significance. The duration of follow-up for each patient and the number of subjects remaining in the study will therefore vary over time. Consequently, the risk of presenting the event of interest will also vary over time and cannot be estimated in the same way as in a binary outcome study with a fixed follow-up period (see above). Survival analysis, adjusted or not, are performed and sometimes a Hazard Ratio (HR) is estimated as well. CVOTs usually report these data.

Thus, the computation of the NNT must take into account the changes in residual risk of the studied population. The NNT is a function of time, whose value varies over time. In other words, there is not a single global NNT representative for one study, but the NNT should be seen as a curve with one value corresponding to each time point t. A NNT at a specific time is interpreted as the average number of patients who will have to be treated to prevent the occurrence of one additional event in the experimental group as compared to the control group at a defined time. Often, the median follow-up time is chosen as the time of particular interest in CVOTs.

In 1999, Altman and Andersen, two renowned statisticians, proposed a method to simply adjust the calculation of NNT for time-to-event studies depending on the type of survival data available (in a publication paper for example) [16]. Basing the calculation on the use of survival analyzes has precisely the advantage of being adjusted according to varying follow-up times and censored data i.e. early dropouts of still “at risk” patients or competing events such as death from another cause than CV.

- Calculating the NNT from survival probabilities of experimental and control groups

If a survival analysis has been performed, an estimate of the survival probability in each group at a given time point should be available and usually in CVOTs, Kaplan–Meier curves have been generated [16, 17]. The NNT is then calculated from the inverse of the survival probability difference between the two groups at a given time t:

$$NNT\left( t \right) = \frac{1}{{{\text{S}}_{exp} \left( {\text{t}} \right) - {\text{S}}_{ctrl} \left( {\text{t}} \right)}}$$

with: S_exp_(t): Probability of event-free survival in the experimental group at time point t; S_ctrl_(t): Probability of event-free survival in the control group at time point t.

Hence, the absolute risk difference is: ARD = S_exp_(t) – S_ctrl_(t).

Regarding the associated 95% CI of the NNT, it might be calculated from the standard error (SE) of each probability of survival by:

$$95\% \; CI\left( {ARD} \right)\mathop \Leftrightarrow \limits_{{}} ARD \pm SE\left( {ARD} \right)\mathop \Leftrightarrow \limits_{{}} \left[ {L_{Lo} ;L_{Up} } \right]$$

Hence: $$95\% \;CI \left( {NNT} \right)\mathop \Leftrightarrow \limits_{{}} \left[ {\frac{1}{{L_{Up} }};\frac{1}{{L_{Lo} }}} \right]$$

with: L_Lo_, the lower limit of the 95% confidence interval; L_Up_, the upper limit of the 95% confidence interval.

b. Calculating the NNT from the Hazard Ratio and the survival probability of the control group

If an adjusted survival analysis has been carried out, an estimate of the probability of survival in each group at a given time point is available, and also an estimate of the Hazard Ratio (HR) [16, 17]. The NNT can therefore be calculated from the Cox regression analysis commonly performed in CVOTs by:

$$NNT\left( t \right) = \frac{1}{{\left[ {{\text{S}}_{ctrl} \left( {\text{t}} \right)} \right]^{HR} {-} {\text{S}}_{ctrl} \left( {\text{t}} \right)}}$$

with: HR: Hazard ratio; S_ctrl_(t): Probability of event-free survival in the control group at time t; [S_ctrl_(t)]^HR^: Probability of event-free survival in the experimental group at time t.

NB: the terms “survival” and “mortality” are often confused in the scientific literature. It is the probability of event-free survival S(t) that must be used in this formula, and not the probability of the event p [17]. As a reminder, note that $S\left( t \right) = 1 - p$

The calculation of the 95% CI of the NNT should be done using the same formula stated above, by simply replacing the value of the HR with the values of its CI.

**Fig. 2:**
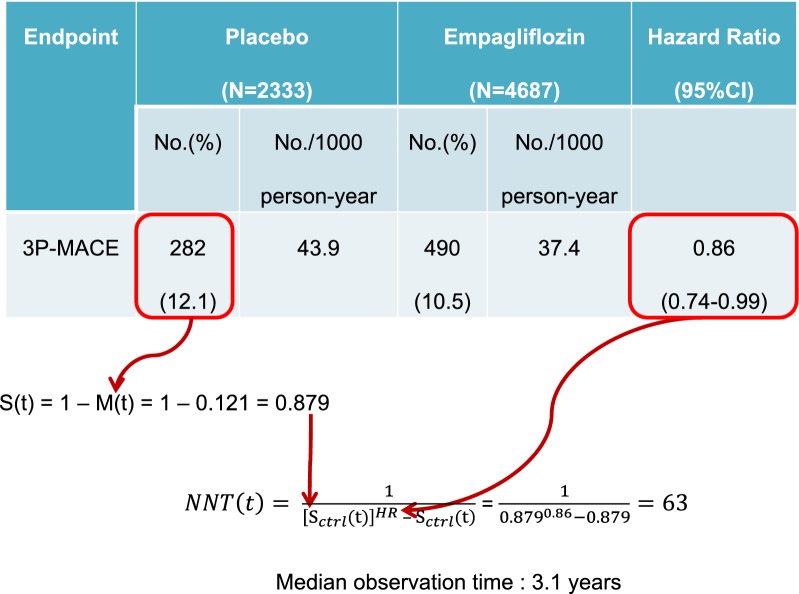
Calculation of the NNT from the EMPAREG-OUTCOME study in practice. N: number of patients; HR: Hazard Ratio; 95% CI: 95% confidence interval; S(t): survival rate at time t; M(t): mortality rate at time t

Let’s take the example of the EMPAREG-OUTCOME study, a CVOT assessing the effect of the SGLT-2 inhibitor empagliflozin on major adverse CV events (3P-MACE) (Fig. 2) [18]. In this trial, 12.1% of patients in the placebo group experienced the primary outcome, which means that 87.9% remained free of event throughout the study. The reported HR was 0.86 (95% CI 0.74–0.99). Using Altman and Andersen’s calculation method, the NNT value for the occurrence of 3P-MACE after a median observation time of 3.1 years in the study would be estimated at 63 patients and the associated 95% CI [34–882]. Beware however that a NNT at 63 does not mean that 1 patient will get the full benefit of the treatment while 62 patients will not benefit at all from it; the benefit might actually be shared by all 63 patients, that is each patient will benefit to some extent from the treatment.

4. Highlight on the confidence interval of the NNT

As with all statistical measures of effect, the uncertainty associated with the measure should be reported through its confidence interval (CI) [14]. As discussed above, NNT CIs values shall be calculated by the reciprocal of the confidence interval of the absolute risk difference depending again on the type of data available. Quite often, these CIs are not reported for NNTs, which may be a concern. Indeed, apparent abnormalities may occur especially when statistically significant difference between the groups has not been reached: if the treatment effect is not significant at the 5% alpha risk as compared to placebo, the 95% CI of the absolute risk difference would include the zero value and therefore, the 95% CI of the NNT would include infinity.

Consider the example of the EXSCEL CVOT, conducted with exenatide extended release [19]. In this trial, 11.4% of patients in the experimental group experienced a 3P-MACE, versus 12.2% in the placebo group. The HR for this comparison was 0.91 (95% CI 0.83–1.00), which was consequently not statistically significant. The NNT value and its 95% confidence interval for the main outcome of the EXSCEL study would therefore be, according to Altman and Andersen’s computation method, as follows:

$$NNT\left( t \right) = \frac{1}{{\left[ {{\text{S}}_{ctrl} \left( {\text{t}} \right)} \right]^{HR} {-} {\text{S}}_{ctrl} \left( {\text{t}} \right)}}\quad {\text{hence}}\quad NNT = \frac{1}{{0.878^{0.91} - 0.878}} = 97.$$

And the 95% CI: $\left[ {\frac{1}{{0.878^{0.83} - 0.878}}; \frac{1}{{0.878^{1} - 0.878}}} \right] = \left[ {51;\infty } \right] .$

The NNT associated with the 3P-MACE of the EXSCEL study with extended release exenatide would then be 97 (95% CI, 51 − $\infty$) after a median follow-up period of 3.2 years. Thus the 95% CI of the NNT might imply that exenatide provides no CV benefit to patients because an infinite number of patients could be treated without avoiding one 3P-MACE. Of course, this result must be weighed with the numerous methodological limitations of the study, which could have minimized the size of the effect (number of study dropouts, more concomitant treatments with CV effectiveness in the placebo group).

## Critical interpretation of NNTs in CVOTs

Through its simplicity and practicality, the NNT has been increasingly used by the scientific community to account for a therapy’s clinical utility. Since 2001, the CONSORT group (CONsolidated Standards Of Reporting Trials) recommends reporting the NNT in the results of randomized controlled trials with binary or time-to-event outcome, in addition to other usual effect measures [20]. However, miscalculations on the one hand and misinterpretation on the other hand may occur while discussing results of CVOTs. Indeed, interpreting an NNT value requires to consider 3 related factors that are not constant across CVOTs: baseline risk, study duration and outcome [21].

**Fig. 3:**
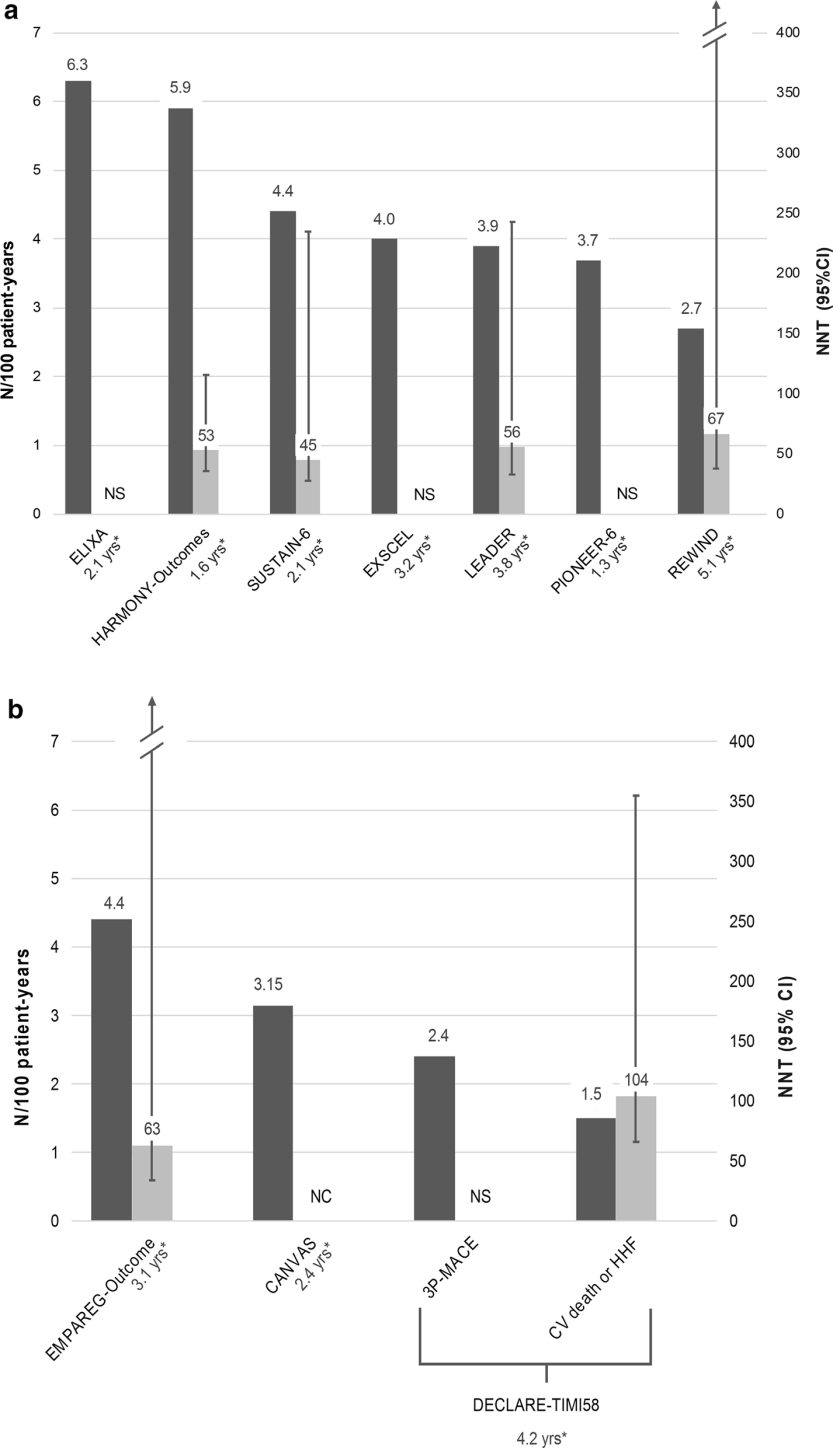
Graphic illustration of annual placebo primary outcome rates and associated NNTs in GLP-1 RA (**a**) and SGLT-2i (**b**) CVOTs. GLP-1 RA: Glucagon Like Peptide-1 receptor agonists; SGLT-2i: Sodium-Glucose Co-Transporter-2 inhibitors; NNT: Number Needed to Treat; CVOTs: cardiovascular outcomes trials; N/100 patient-years: number per 100 patient-years; 95% CI: 95% confidence interval; CV: cardiovascular; HHF: hospitalization for heart failure; NS: not significant; NC: not calculable because required data for calculation were not available in the publication paper or supplementary appendix. *median study follow-up in years; Primary outcome was a 3-points MACE (Major Adverse Cardiovascular Events) for all studies, except ELIXA (4-points MACE) and DECLARE-TIMI58 (co-primary endpoint: 3P-MACE and CV death or HHF); Dark grey bars represent annual placebo primary outcome rates; Light grey bars represent NNTs with 95% CI; regarding data from the REWIND and EMPAREG-Outcome studies, a vertical arrow and 2 slash signs were used to represent the upper limit of their respective 95% confidence intervals for NNTs on a sensible scale

The first factor to consider is the baseline risk of the studied population. Indeed, the NNT will inversely vary with the baseline risk, which means that if the baseline risk of a study population is low, one should expect a greater NNT. In most CVOTs, populations with high or very high CV risk were selected, in order to ensure a high absolute risk and therefore a high probability of CV events over a short time period (Table 1; Fig. 3) [18, 19, 22–29]. For example, in the HARMONY-Outcomes study evaluating the CV safety of albiglutide, all patients had very high CV risk or established CV disease, as reflected by the annual placebo primary outcome rate of 5.9 per 100 patient-year [24]. The NNT associated with the 3P-MACE, was 53 (95% CI, 36–116) after a median duration of follow-up of 1.6 years. In contrast, patients enrolled in the REWIND study with dulaglutide were overall at a lower CV risk as evidenced by the annual placebo primary outcome rate of 2.7 per 100 patient-year, even though the proportion of patients at very high and high CV risk is unknown [25, 30]. The NNT associated with 3P-MACE was 67 (95% CI, 38–803) with a median follow-up of 5.4 years. Given the large difference in the absolute risk level of these two populations at baseline (HARMONY-Outcomes and REWIND), considering an indirect comparison of the two drugs and seeking establishing a power ranking between albiglutide and dulaglutide based on respective NNTs, would be highly inappropriate (or even wrong).

**Table 1:** Summary patient and study characteristics influencing the NNT value in GLP-1 RA and SGLT-2i CVOTs

CVOT	Drug	Primary outcome	Annual placebo primary outcome rate (N/100 patient-year)	Median follow-up (years)	NNT (according to Altman & Andersen’s formula) with 95% CI
*GLP*-*1 RA*					
LEADER [22]	Liraglutide	3P-MACE	3.9	3.8	56 [33–243]
SUSTAIN-6 [23]	Semaglutide	3P-MACE	4.4	2.1	45 [28–235]
HARMONY-Outcomes [24]	Albiglutide	3P-MACE	5.9	1.6	53 [36–116]
REWIND [25]	Dulaglutide	3P-MACE	2.7	5.1	67 [38–803]
EXSCEL [19]	Exenatide	3P-MACE	4.0	3.2	Not significant
ELIXA [26]	Lixisenatide	4P-MACE	6.3	2.1	Not significant
PIONEER-6 [27]	Semaglutide (oral)	3P-MACE	3.7	1.3	Not significant
*SGLT*-*2i*					
EMPAREG-Outcome [18]	Empagliflozin	3P-MACE	4.4	3.1	63 [34–882]
DECLARE-TIMI58 [28]	Dapagliflozin	CV death or hospitalization for heart failure	1.5	4.2	104 [66–355]
		3P-MACE	2.4	4.2	Not significant
CANVAS [29]	Canagliflozin	3P-MACE	3.15	2.4	Not calculable*

*CVOT* Cardiovascular Outcomes Trial, *CV* cardiovascular, *3P-MACE* 3 points Major Adverse Cardiovascular Events

*Required data for calculation were not available in the publication paper or supplementary appendix

The second factor that must be taken into account is the duration of the study. Each NNT is associated to a specific duration, usually the median follow-up time point. A certainly tempting error would be to seek to standardize study follow-up durations to be able to compare NNTs on a standardized time period [7, 21]. For example, one could imagine converting each specific NNTs of each CVOTs into a standardized 1-year period of follow-up. Again, this would be incorrect because when the follow-up duration increases, the NNT will accordingly tend to decrease since the absolute event rate gets higher. However, such projections to different time frames have been proposed, for instance with ARNI on the basis of data from the PARADIGM-HF trial (27 months median follow-up) in order to estimate the 5-year NNT [10]. Despite the use of a sophisticated statistical model, data generated should be considered as exploratory and take the limitations underlined by the authors into account. Besides, CVOTs are typically long duration studies, which could potentially leave competing events, such as a death from another cause, come into play and influence the occurrence of the event of interest [31]. Thus, as NNT values will vary non-linearly over time, extrapolating some NNT results to a different time horizon, shorter or longer, would be inappropriate. It is common sense for any clinician to say that treating 60 patients for 3 years would not be as effective as treating 180 patients for 1 year.

And thirdly, the outcome itself plays a role. A NNT is specific to a defined study endpoint, so that the NNT of each endpoint of interest should be taken into account to interpret the overall benefit/risk balance of a treatment Take the example of the DECLARE-TIMI58 study with dapagliflozin designed with two co-primary endpoints: a 3P-MACE and a composite of CV mortality and hospitalization for heart failure [28]. The associated NNT were respectively, 160 and 104 after 4.2 years of treatment (Note: the comparison of the two groups regarding the 3P-MACE endpoint was not significant, which questions the interpretation of the associated NNT CI. See previous section). Finally, one might also imagine calculating a NNT for safety parameters in addition to the efficacy ones. In the example of the DECLARE-TIMI58 study, a serious adverse event would occur every 38 patients treated.

In conclusion, an NNT should not be considered as an absolute measure of the overall clinical benefit of a treatment. A difference in NNT across trials may be attributable to a true difference in treatment effectiveness as much as a difference in patients’ baseline risk or any other CVOT characteristic. Further, the treatment benefit based on an estimated NNT should be, as always, weighed with its toxicity, and possibly its cost for an overall assessment of the drug’s efficiency [32]. Finally, indirect comparisons between NNTs of two separate studies should be avoided since they are not adjusted in the same way [21].

## Conclusion

Given the great medical impact of CVOTs, a correct calculation of the NNT is a fundamental point, and the first step to an appropriate interpretation. In the absence of cardio-renal events studies conducted under the same conditions, there is to date no justification for establishing a strong ranking of power or efficiency between the different GLP-1 RA and SGLT-2i based on NNTs. However, NNTs could provide input to the design of a medico-economic model taking efficacy and safety parameters as well as cost into account, in order to have a full picture of these drugs cost-effectiveness.

## Data Availability

Not applicable.

## Abbreviations

ADA/EASD: American Diabetes Association/European Association for the Study of Diabetes
ARD: Absolute risk difference
ARNI: Angiotensin receptor–neprilysin inhibitor
CV: Cardiovascular
CVD: Cardiovascular disease
CVOT: Cardiovascular Outcomes Trial
ESC: European Society of Cardiology
GLP-1 RA: Glucagon-like-peptide receptor agonist
HR: Hazard ratio
NNT: Number Needed to Treat
PCSK-9: Proprotein convertase subtilsin-kexin type 9
RCT: Randomized controlled trial
SGLT-2i: Sodium-glucose co-transporter-2 inhibitors
T2D: Type 2 diabetes
3P-MACE: 3-Point major adverse cardiovascular events
